# Interaction of 2,4-D or Dicamba with Glufosinate for Control of Glyphosate-Resistant Giant Ragweed (*Ambrosia trifida* L.) in Glufosinate-Resistant Maize (*Zea mays* L.)

**DOI:** 10.3389/fpls.2017.01207

**Published:** 2017-07-10

**Authors:** Zahoor A. Ganie, Amit J. Jhala

**Affiliations:** Department of Agronomy and Horticulture, University of Nebraska-Lincoln, LincolnNE, United States

**Keywords:** glyphosate resistant, herbicide interaction, tank-mixture, weed control

## Abstract

Glyphosate-resistant (GR) giant ragweed is a problematic broadleaf weed in crops including maize and soybean in the Midwestern United States. Commercialization of crops with 2,4-D or dicamba and glufosinate resistance will allow post-emergence (POST) applications of these herbicides. Therefore, information is needed on how 2,4-D/dicamba will interact with glufosinate in various rate combinations. The objectives of this study were to evaluate the interaction of glufosinate plus 2,4-D and/or dicamba for control of GR giant ragweed, and to determine their effect on GR giant ragweed density, biomass, maize injury, and yield. Field experiments were conducted in 2013 and 2014 in a field infested with GR giant ragweed in Nebraska, United States. The treatments included POST applications of glufosinate (450 or 590 g ai ha^-1^), 2,4-D, or dicamba at 280 or 560 g ae ha^-1^ applied alone and in tank-mixtures in glufosinate-resistant maize. The results showed that dicamba applied alone resulted in 56 to 62% and 73 to 83% control at 14 and 28 days after treatment (DAT), respectively, and ≥95% control at 60 DAT or at harvest compared to 17 to 30% and 57 to 73% control with 2,4-D applied alone at 280 and 560 g ai ha^-1^, respectively. Glufosinate tank-mixed with 2,4-D and/or dicamba consistently provided ≥89% control of GR giant ragweed, except that control with glufosinate plus 2,4-D varied from 80 to 92% at 60 DAT and at harvest. The comparison between the observed and expected control (determined by Colby’s equation) suggested an additive interaction between glufosinate and 2,4-D or dicamba for control of GR giant ragweed. Contrast analysis also indicated that GR giant ragweed control with glufosinate plus 2,4-D or dicamba was either consistently higher or comparable with individual herbicides excluding 2,4-D applied alone. Herbicide programs, excluding 2,4-D at 280 g ae ha^-1^, resulted in ≥80% reduction in GR giant ragweed density. Tank-mixing glufosinate with 2,4-D or dicamba showed an additive effect and will be an additional tool with two effective modes of action for the management of GR giant ragweed in maize.

## Introduction

Multiple herbicide-resistant crops, such as 2,4-D or dicamba plus glyphosate and/or glufosinate-resistant soybean, will be planted in the United States in the near future (Green, 2016). This technology will provide an additional tool for the management of glyphosate-resistant (GR) weeds (Diggle et al., 2003; Green et al., 2008; Vink et al., 2012; Craigmyle et al., 2013a,b). Moreover, new herbicides recently registered or labeled in maize and soybean are pre-mixtures of existing herbicides with multiple effective modes of action (Chahal et al., 2015; Ganie et al., 2015; Sarangi and Jhala, 2017). Herbicide pre-mixtures or tank-mixtures are typically based on the assumption that any rare individual in a weed population naturally insensitive or less sensitive to one herbicide active ingredient in an herbicide mixture should not be able to express a fitness advantage and survive in the presence of additional effective herbicide active ingredient(s) (Lagator et al., 2013). Therefore, herbicide active ingredients with different modes of action present in an herbicide mixture should have a common weed control spectrum, similar efficacy, and persistence, along with different metabolic pathways to effectively reduce the selection pressure and delay the evolution of herbicide-resistant weeds (Wrubel and Gressel, 1994). The commercialization of multiple herbicide-resistant crops will increase the use of herbicide mixtures with auxinic herbicides (2,4-D or dicamba) plus glufosinate to effectively control weeds, including GR weeds, in maize, soybean [*Glycine max* (L.) Merr.], and cotton in the United States (*Gossypium hirsutum* L.) (Vink et al., 2012; Craigmyle et al., 2013a,b; Merchant et al., 2013).

Synthetic auxin herbicides such as 2,4-D and dicamba are important systemic herbicides for the control of broadleaf weeds (Vink et al., 2012; Peterson et al., 2016). Synthetic auxin herbicides cause an up-regulation of auxin responses in plants leading to a disturbance in the balance of natural plant growth hormones that interrupts normal growth and differentiation; triggers abnormal unregulated cell division; causes uncontrolled growth; and causes damage to chloroplasts, membranes, and vascular tissues (Grossmann, 2010). Dicamba and 2,4-D have been successfully used for over 40 and 70 years, respectively, to control broadleaf weeds primarily in cereal crops and non-crop areas (Behrens et al., 2007; Peterson et al., 2016). The recent development of 2,4-D and dicamba-resistant crops will provide an opportunity to apply these herbicides post-emergence (POST) (Vink et al., 2012; Craigmyle et al., 2013a,b; Merchant et al., 2013). As of January 2017, 18 and 6 weed species worldwide have evolved resistance to 2,4-D and dicamba, respectively, including three species resistant to 2,4-D and two species resistant to dicamba in the United States (Heap, 2017).

Glufosinate is an important broad-spectrum contact herbicide that can be used in tank-mixture with 2,4-D, dicamba and/or glyphosate in the newly developed multiple herbicide-resistant maize, soybean, and cotton (Vink et al., 2012; Barnett and Steckel, 2013; Craigmyle et al., 2013a,b; Merchant et al., 2013). Glufosinate inhibits the activity of glutamine synthetase, an enzyme responsible for the synthesis of glutamine from glutamate plus ammonia, resulting in the buildup of ammonia in cells and the disruption of the plant’s nitrogen metabolism (Wendler et al., 1990; Wild and Wendler, 1991). Toxic concentration of ammonia in the cells usually disrupts the cell’s chloroplast structure, prevents normal photosynthesis and photophosphorylation, and eventually destroys the cells (Devine et al., 1993; Hinchee et al., 1993). Glufosinate is effective for the control of a wide spectrum of weeds, including broadleaf and grass weeds (Steckel et al., 1997). Additionally, glufosinate is also effective for control of certain weed species such as morningglory (*Ipomoea* spp.) and hemp sesbania [*Sesbania exaltata* (Raf.) Rydb. ex A.W. Hill], which are naturally less sensitive to glyphosate (Corbett et al., 2004). Previous studies have reported that synthetic auxin herbicides (2,4-D or dicamba) plus glufosinate provided effective control of broadleaf weeds: for example, Palmer amaranth [*Amaranthus palmeri* (L.) Wats.] control improved from 89 to 97% with glufosinate plus 2,4-D or dicamba compared to less than 83% control with the respective herbicides applied alone (Merchant et al., 2013). Craigmyle et al. (2013b) reported that control of Asiatic dayflower (*Commelina communis* L.) and common waterhemp (*Amaranthus rudis* Sauer) was 68 to 92% and more than 95%, respectively, with a tank-mixture of glufosinate and 2,4-D compared to glufosinate or 2,4-D applied alone (0 to 22% and 75 to 95% control, respectively).

Giant ragweed (*Ambrosia trifida* L.) is an important summer annual broadleaf weed found in wastelands, roadsides, fence-lines, and agronomic crops including maize, soybean, and cotton (Abul-Fatih and Bazzaz, 1979; Bassett and Crompton, 1982; Johnson et al., 2006). Giant ragweed has a competitive advantage over crops and other annual weed species due to its early emergence, large leaf area, rapid growth rate, high plasticity, and ability to regulate its resource utilization in response to changing environmental factors (Abul-Fatih and Bazzaz, 1979; Bazzaz and Carlson, 1979). Management of giant ragweed has become complicated due to its extended emergence pattern in the eastern maize belt of the United States (Schutte et al., 2008, 2012) and due to the evolution of giant ragweed biotypes resistant to ALS-inhibitors and/or glyphosate in the Midwestern United States (Johnson et al., 2006; Norsworthy et al., 2010, 2011; Jhala et al., 2014; Kaur et al., 2014). Nevertheless, effective integrated management options for GR giant ragweed based on preplant tillage followed by PRE and/or POST applications of herbicide-mixtures have been reported in maize and soybean (Ganie et al., 2016, 2017). Recent research in Nebraska has reported that giant ragweed is sensitive to synthetic auxin herbicides and can be effectively controlled by a preplant application of 2,4-D (Jhala et al., 2014; Kaur et al., 2014; Ganie et al., 2016). Similarly, glufosinate plus 2,4-D or dicamba provided greater than 90% control of GR giant ragweed (Barnett and Steckel, 2013). Vink et al. (2012) reported 100% control of GR giant ragweed with preplant followed by POST applications of glyphosate plus dicamba in dicamba-tolerant soybean.

Use of herbicide tank-mixtures or pre-mixtures has become a common reactive approach for the management of herbicide-resistant weeds (Green, 1991; Buttel, 2002; Hart and Pimentel, 2002; Beckie and Reboud, 2009). Typically, herbicide mixtures that provide improved weed control while allowing a reduced dose of the component herbicides are considered economically viable (Gressel, 1993). However, reduced doses of the component active ingredients in an herbicide mixture may affect synergistic interactions and result in disproportionate performance of the component herbicides (Green, 1991). In addition, the exposure of weed populations to the lower herbicide doses usually used in herbicide mixtures may result in selection for generalist type mutation(s) or non-target site mechanisms, providing resistance to all the herbicide active ingredients present in the mixture (Neve and Powles, 2005). Relatively large differences in the efficacy of the component herbicide in a mixture exposes weed population to a higher selection pressure of the better performing partner, and likely reduce the potential of the herbicide tank-mixture to delay the evolution of herbicide resistance (Beckie and Reboud, 2009).

Previous research has shown that a specific rate of the constituent active ingredients is needed for herbicide mixtures with synergistic interaction. For example, Hugie et al. (2008) reported that a threshold mesotrione rate was needed to attain synergism between mesotrione and atrazine. Therefore, research is needed to determine the effect of individual herbicide rates on the type of interaction (additive, synergistic, or antagonistic) between glufosinate with 2,4-D and/or dicamba. The objectives of this study were to evaluate the efficacy of 2,4-D and/or dicamba plus glufosinate for control of GR giant ragweed, and to determine the effect of herbicide rate combinations on the type of interaction between 2,4-D and/or dicamba plus glufosinate on giant ragweed control, density, biomass, maize injury, and yield. We hypothesized that an additive interaction between synthetic auxins (2,4-D or dicamba) and glufosinate for GR giant ragweed control will be achieved when constituent herbicides are used at the labeled rate in tank-mixture.

## Materials and Methods

### Field Experiments

Field experiments were conducted at Clay Center (40.52°N, 98.05°W) and David City (41.25°N, 97.13°W), Nebraska in 2013 and 2014, respectively, in growers’ fields infested with GR giant ragweed. Giant ragweed biotypes from these sites were confirmed resistant to glyphosate in 2011, with 14-fold resistance compared to glyphosate-susceptible biotypes included for comparison (Rana et al., 2013). The density of GR giant ragweed at these sites varied from 18 to 30 plants m^-2^. The soil type at Clay Center was fine, smectitic, mesic Vertic Argiaquolls (Butler series) with a silt loam texture (17% sand, 58% silt, 25% clay), 2.5% organic matter, and a pH of 6.5. The soil type at David City was fine, smectitic, mesic Udic Argiustolls (Hastings series) with a silty clay loam texture (18% sand, 50% silt, 32% clay), 2.1% organic matter, and a pH of 5.4. The experiment was arranged in a randomized complete block design with 18 treatments and four replications. The treatments included POST applications of glufosinate (450 or 590 g ai ha^-1^) (Liberty 280, Bayer Crop Science, Research Triangle Park, NC, United States), 2,4-D amine (280 or 560 g ae ha^-1^) (2,4-D Amine, Winfield Solutions, LLC, St Paul, MN, United States), and dicamba (280 or 560 g ae ha^-1^) (Clarity, BASF Corporation, Research Triangle Park, NC, United States) alone and in tank-mixtures (**Table 1**). Treatment with no herbicide application served as a non-treated control for comparison. Maize seeds (Cv. “Pioneer 1151AM” in 2013 and “Mycogen 2V709” in 2014) with resistance to both glyphosate and glufosinate were planted on May 16, 2013 and May 17, 2014. The seeds were planted 3 cm deep at a density of 79,000 seeds ha^-1^. Individual plots were 3 m wide and 9 m long with four maize rows spaced 76 cm apart.

**Table 1:** Observed and expected control of glyphosate-resistant giant ragweed with 2,4-D, dicamba, and glufosinate applied alone or in tank-mixtures in glyphosate plus glufosinate-resistant maize in field experiment conducted in 2013 and 2014 in Nebraska, United States.^a,b^

Herbicide treatment	Rate	Giant ragweed control^c^				Expected control based on Colby’s equation^d,e^	
		14 DAT	28 DAT	60 DAT	At harvest	14 DAT	60 DAT
	g ae or ai ha^-1^	^_________________________________________^ % ^_________________________________________^					
Non-treated control	–	0	0	0	0	–	–
2,4-D	280	30 d	20 f	17 e	18 f	–	–
2,4-D	560	57 c	73 e	71 d	66 e	–	–
Dicamba	280	56 c	73 e	97 ab	95 abc	–	–
Dicamba	560	62 c	83 de	99 a	99 a	–	–
Glufosinate	450	92 ab	89 bcd	70 d	79 cde	–	–
Glufosinate	590	87 b	85 dc	83 bcd	87 abcde	–	–
Glufosinate + 2,4-D	450 + 280	96 a	91 abcd	80 dc	81 cde	94	75
Glufosinate + 2,4-D	450 + 560	95 a	93 abc	92 abcd	91 abcd	97	91
Glufosinate + 2,4-D	590 + 280	94 ab	91 abcd	80 dc	84 bcde	91	86
Glufosinate + 2,4-D	590 + 560	93 ab	93 abc	82 bcd	86 bcde	94	95
Glufosinate + dicamba	450 + 280	95 a	96 a	89 abcd	92 abcd	96	99
Glufosinate + dicamba	450 + 560	95 a	96 a	97 ab	98 ab	97	100
Glufosinate + dicamba	590 + 280	95 a	95 ab	97 ab	92 abcd	94	100
Glufosinate + dicamba	590 + 560	96 a	97 a	94 abc	95 abc	95	100
Dicamba + 2,4-D	280 + 140	66 c	84 de	94 abc	98 ab	–	–
Glufosinate + dicamba + 2,4-D	450 + 280 + 140	90 ab	93 abc	90 abcd	94 abc	99	98
Glufosinate + dicamba + 2,4-D	590 + 280 + 140	92 ab	94 ab	92 abcd	94 abc	96	99
*p*-value		<0.0001	<0.0001	<0.0001	<0.0001		

*^a^Abbreviation: DAT, days after treatment. ^b^Data from non-treated control plots were excluded from the analysis. ^c^Means followed by the same letter within a column are not statistically different according to Fisher’s protected LSD at *P* ≤ 0.05. ^d^Expected values of giant ragweed control were determined by the Colby equation: $\begin{array}{c} E=(X+Y)-\left(\frac{XY}{100}\right) \end{array}$, where *E* is the expected control of giant ragweed with application of herbicides A + B in tank-mixture, and *X* and *Y* are the observed control with the application of herbicides A and B, respectively, at specific rates. ^e^The observed and expected control at 14 and 60 DAT were compared using a *t*-test that suggested no statistical differences, indicating that 2,4-D and/or dicamba showed an additive interaction with glufosinate.*

Herbicide treatments were applied as POST (June 5, 2013 and June 6, 2014) on 8 to 12 cm tall (4 to 6 leaf stage) giant ragweed plants. Herbicides were applied with a CO_2_-pressurized backpack sprayer equipped with a four-nozzle boom fitted with XR11015 flat-fan nozzles (TeeJet, Spraying Systems Co., Wheaton, IL, United States) and calibrated to deliver 140 L ha^-1^ at 276 kPa. The experimental location was under rainfed conditions in 2013 and irrigated conditions in 2014.

### Data Collection

Data were collected for visual assessments of giant ragweed control with treatments compared to non-treated control on a scale of 0 to 100% (0 being no control and 100 being complete control) at 14, 28, and 60 days after POST herbicide treatments (DAT), and before maize harvest. Herbicide-injury symptoms including slight bending of the maize plants with 2,4-D or dicamba, and chlorotic spots characteristic of glufosinate on maize canopy were recorded on a scale of 0 to 100% (0 being no injury and 100 being plant death) at 14 and 21 DAT. Giant ragweed density was recorded from three randomly selected 0.25 m^2^ quadrats per plot at 60 DAT. Glyphosate-resistant giant ragweed biomass was assessed from three randomly selected 0.25 m^2^ quadrats per plot at 60 DAT. Giant ragweed plants that survived herbicide treatments were cut at the stem base close to the soil surface, placed in paper bags, dried in an oven for 72 h at 50°C, and weighed (g). Maize was harvested using a plot combine and yields were adjusted to 15% moisture content (Harrison et al., 2001). Giant ragweed biomass data were converted into percent biomass reduction compared to the non-treated control (Sarangi et al., 2017) as:

(1) $$\begin{array}{c} \mathrm{Biomass}\mathrm{reduction}(\%)\;=\left[\frac{\left(C-B\right)}{C}\right]\times 100 \end{array}$$

where *C* is the biomass of the non-treated control replicates and *B* is the biomass of an individual treated experimental unit.

### Statistical Analysis

Data were subjected to ANOVA using the PROC GLIMMIX procedure in SAS version 9.3 (SAS Institute Inc, Cary, NC, United States). The treatments with zero response variables were not included in the analyses. Before analyses, data were tested for normality of residuals using the PROC UNIVARIATE procedure in SAS, which suggested that data does not follow a Gaussian distribution. Therefore, visual estimates of giant ragweed control, and biomass reduction data were arcsine square-root transformed before analysis; however, back-transformed data are presented with mean separation based on the transformed data. When the ANOVA indicated that treatment effects were significant, means were separated at *P* ≤ 0.05 using Fisher’s protected least significant difference (LSD) test. Single degree-of-freedom contrast statements were used to compare herbicide programs with 2,4-D, dicamba, or glufosinate applied alone vs. their tank-mixtures. Specific contrast statements were used to compare 2,4-D vs. dicamba, glufosinate plus 2,4-D or dicamba vs. alone application of these herbicides, and glufosinate plus 2,4-D plus dicamba vs. glufosinate plus 2,4-D or dicamba. To determine the type of interaction (additive, synergistic, or antagonistic) between herbicide programs, the Colby equation was used to calculate the expected values (Colby, 1967):

(2) $$\begin{array}{c} E=(X+Y)-\left(\frac{XY}{100}\right) \end{array}$$

where *E* is the expected control of giant ragweed with application of herbicides A + B in tank-mixture, and *X* and *Y* are the observed control with the application of herbicides A and B, respectively, at specific rates. The statistical differences between the expected and observed values of control were determined by the *t*-test in R (R statistical software, R Foundation for Statistical Computing, Vienna, Austria^1^). The herbicide combination was considered synergistic if the expected mean was significantly lower than the observed mean. If the expected mean was greater than the observed mean, the herbicide combination was considered antagonistic (Colby, 1967).

## Results

Year-by-treatment interactions for visual estimates of giant ragweed control, density, and aboveground biomass reduction were not significant (*P* ≥ 0.05); therefore, data were combined over 2 years. However, year-by-treatment interaction for maize yield was significant; therefore, yield is presented separately for both years.

### Giant Ragweed Control

The application of 2,4-D at 280 and 560 g ae ha^-1^ resulted in 30 and 57% control of GR giant ragweed at 14 DAT, respectively (**Table 1**). However, dicamba resulted in comparable giant ragweed control (56 to 62%) with both rates (280 and 560 g ae ha^-1^) (**Table 1**). Averaged across application rates, GR giant ragweed control with 2,4-D was 44% compared to 59% control with dicamba (**Table 2**). In contrast, GR giant ragweed control with glufosinate at 450 or 560 g ai ha^-1^ was 87 to 92% (**Table 1**). Herbicide programs including glufosinate plus 2,4-D or dicamba, and glufosinate plus dicamba plus 2,4-D tank-mixed at various rates provided ≥90% giant ragweed control in contrast to 66% control with dicamba plus 2,4-D at 14 DAT (**Table 1**).

**Table 2:** Contrast statements to compare herbicide programs for control of glyphosate-resistant giant ragweed in glyphosate plus glufosinate-resistant maize in a field experiment conducted in 2013 and 2014 in Nebraska, United States.^a,b^

Treatment	Giant ragweed control^c^ (%)			
	14 DAT	28 DAT	60 DAT	At harvest
2,4-D vs. dicamba	44 vs. 59^∗∗^	47 vs. 78^∗∗^	44 vs. 98^∗∗^	42 vs. 97^∗∗^
Glufosinate + 2,4-D vs. 2,4-D alone	95 vs. 44^∗∗^	92 vs. 47^∗∗^	84 vs. 44^∗∗^	86 vs. 42^∗∗^
Glufosinate + 2,4-D vs. glufosinate alone	95 vs. 90^∗^	92 vs. 87 NS	84 vs. 77 NS	86 vs. 83 NS
Glufosinate + dicamba vs. dicamba alone	95 vs. 59^∗∗^	96 vs. 78^∗∗^	94 vs. 98 NS	94 vs. 97 NS
Glufosinate + dicamba vs. glufosinate alone	95 vs. 90^∗^	96 vs. 87^∗∗^	94 vs. 77^∗∗^	94 vs. 83^∗^
Glufosinate + 2,4-D + dicamba vs. glufosinate + 2,4-D	91 vs. 95 NS	94 vs. 92 NS	91 vs. 84^∗^	94 vs. 86^∗^
Glufosinate + 2,4-D + dicamba vs. glufosinate + dicamba	91 vs. 95 NS	94 vs. 96 NS	91 vs. 94 NS	94 vs. 94 NS

*^a^Preplanned single degree of freedom contrast statements were performed to compare treatments with herbicides used alone (2,4-D, dicamba or glufosinate) versus treatment combinations of 2,4-D and/or dicamba plus glufosinate, and three-way versus two-way combinations of 2,4-D, dicamba, and glufosinate. ^b∗^Significant with *P* ≤ 0.05; ^∗∗^significant with *P* ≤ 0.01, NS, non-significant or *P* ≥ 0.05. ^c^Abbreviation: DAT, days after treatment.*

Herbicide programs excluding 2,4-D at 280 g ae ha^-1^ resulted in ≥73% GR giant ragweed control at 28 DAT; for example, dicamba at 560 g ae ha^-1^ and glufosinate at 450 or 590 g ai ha^-1^ resulted in 83 to 89% control of GR giant ragweed. At 28 DAT, control with 2,4-D plus dicamba improved to 84% compared to the previous rating (66%), though ≥91% control was achieved with glufosinate plus 2,4-D or dicamba (**Table 1**). Irrespective of the individual herbicide rate in the tank-mixtures evaluated in this study, glufosinate plus dicamba provided 95 to 97% control compared to 91 to 93% control with glufosinate plus 2,4-D (**Table 1**). Similarly, the contrast analysis suggested that control of GR giant ragweed with glufosinate plus 2,4-D or dicamba was greater compared to glufosinate, 2,4-D or dicamba applied alone at 14 and 28 DAT except that the contrast between glufosinate plus 2,4-D vs. glufosinate was not significant (*P* > 0.05) at 28 DAT (**Table 2**).

Control of GR giant ragweed at 60 DAT and at harvest was less than or equal to 18% with 2,4-D applied alone at 280 g ae ha^-1^ compared to 66 to 71% control when applied at 560 g ae ha^-1^ (**Table 1**). Irrespective of the application rate, dicamba provided an effective control of GR giant ragweed ranging from 95 to 99% at 60 DAT or at harvest (**Table 1**). Giant ragweed control improved from 70 to 79% with glufosinate applied at 450 g ai ha^-1^ to 83 to 87% control at 590 g ai ha^-1^ (**Table 1**). Moreover, tank-mixing glufosinate with dicamba resulted in 89 to 98% giant ragweed control compared to glufosinate plus 2,4-D (80 to 92%), with limited difference among treatments (**Table 1**). For example, glufosinate (450 or 590 g ai ha^-1^) plus 2,4-D (280 g ae ha^-1^) resulted in 80% giant ragweed control compared to 97% control with glufosinate at 450 or 590 g ai ha^-1^ tank-mixed with dicamba at 560 or 280 g ae ha^-1^, respectively, at 60 DAT (**Table 1**). In contrast, three way tank-mixtures of glufosinate plus dicamba plus 2,4-D provided comparable control of giant ragweed at 60 DAT or at harvest ranging from 90 to 94% regardless of glufosinate application rate (**Table 1**). However, the contrast analysis showed that giant ragweed control with glufosinate plus 2,4-D, glufosinate plus dicamba, and glufosinate plus 2,4-D plus dicamba was better compared to 2,4-D, glufosinate, and glufosinate plus 2,4-D, respectively, at 60 DAT or at harvest (**Table 2**). Additionally, the contrasts between 2,4-D vs. dicamba were significant (*P* ≤ 0.01) indicating that dicamba provided greater GR giant ragweed control compared to 2,4-D (**Table 2**).

The expected values of giant ragweed control for herbicide mixtures including glufosinate plus 2,4-D and/or dicamba at 14 and 60 DAT determined by Colby’s equation were not different compared to observed values (**Table 1**), indicating that tank-mixtures of glufosinate plus 2,4-D and/or dicamba at the rates used in this study showed an additive interaction for control of GR giant ragweed.

### Giant Ragweed Density and Aboveground Biomass Reduction

Glyphosate-resistant giant ragweed density and aboveground biomass reduction were in consensus with the visual assessment of control at 60 DAT (**Tables 1, 3**). The highest giant ragweed density with an average of 20 plants m^-2^ was recorded in the untreated control. Among herbicide programs, the highest giant ragweed density with an average of 11 plants m^-2^ was observed with 2,4-D applied at 280 g ae ha^-1^, while the remaining treatments resulted in ≥80% reduction in giant ragweed density (2 to 4 plants m^-2^), including a 100% reduction with dicamba at 560 g ae ha^-1^ (**Table 3**). Among herbicide treatments, the contrast analysis of GR giant ragweed density indicated differences (*P* ≤ 0.05) only between 2,4-D (8 plants m^-2^) vs. dicamba (2 plants m^-2^), and glufosinate plus 2,4-D (3 plants m^-2^) vs. 2,4-D (8 plants m^-2^) (**Table 4**). Among all herbicide programs, the lowest aboveground biomass reduction was 38% with 2,4-D at 280 g ae ha^-1^ (**Table 3**). Most of the herbicide treatments resulted in ≥80% reduction in aboveground biomass of GR giant ragweed, with the exception of 68, 74, and 78% biomass reduction with glufosinate (590 g ai ha^-1^) plus 2,4-D (560 g ae ha^-1^), glufosinate (590 g ai ha^-1^), and glufosinate (450 g ai ha^-1^) plus 2,4-D (280 g ae ha^-1^), respectively (**Table 3**). Similarly, contrast statements for the aboveground biomass reduction were significant (*P* ≤ 0.05) only between 2,4-D vs. dicamba and glufosinate plus 2,4-D vs. 2,4-D, and glufosinate plus dicamba vs. glufosinate (**Table 4**).

**Table 3:** Glyphosate-resistant giant ragweed density, aboveground biomass reduction, and corn yield affected by 2,4-D, dicamba, and glufosinate applied alone or in tank-mixtures in glyphosate plus glufosinate-resistant maize in a field experiment conducted in 2013 and 2014 in Nebraska, United States.^a^

Herbicide	Rate	Giant ragweed^b^		Maize yield^b^	
		Density	Aboveground biomass reduction	2013	2014
	g ae or ai ha^-1^	No. m^-2^	%	^________________^kg ha^1_________________^	
Non-treated Control	–	20 a	0	0	0
2,4-D	280	11 b	38 e	5,23 d	6,280 g
2,4-D	560	4 c	88 abc	6,715 abc	10,205 abc
Dicamba	280	3 c	89 abc	6,211 abc	11,018 ab
Dicamba	560	0	100 a	9,924 abc	11,554 ab
Glufosinate	450	4 c	81 abcd	6,785 abc	7,246 dc
Glufosinate	590	4 c	74 dc	5,903 abc	10,143 abc
Glufosinate + 2,4-D	450 + 280	3 c	78 bcd	5,035 dc	11,018 ab
Glufosinate + 2,4-D	450 + 560	3 c	91 abc	6,519 abc	10,845 ab
Glufosinate + 2,4-D	590 + 280	3 c	84 abcd	5,066 bcd	8,821 bcd
Glufosinate + 2,4-D	590 + 560	4 c	68 d	5,745 abcd	11,143 ab
Glufosinate + dicamba	450 + 280	2 c	91 abc	9,057 abc	9,200 bcd
Glufosinate + dicamba	450 + 560	2 c	95 ab	10,783 a	12,416 a
Glufosinate + dicamba	590 + 280	2 c	86 abcd	8,808 abc	10,730 ab
Glufosinate + dicamba	590 + 560	3 c	95 ab	10,347 ab	11,030 ab
Dicamba + 2,4-D	280 + 140	2 c	92 abc	8,424 abc	11,014 ab
Glufosinate + dicamba + 2,4-D	450 + 280 + 140	4 c	85 abcd	7,196 abc	9,861 abc
Glufosinate + dicamba + 2,4-D	590 + 280 + 140	2 c	82 abdc	7,129 abc	10,425 abc
*p*-value		<0.0001	<0.0001	<0.0696	<0.0001

*^a^Data from non-treated control plots were excluded from the analysis. ^b^Means followed by the same letter within a column are not statistically different according to Fisher’s protected LSD test at *P* ≤ 0.05.*

**Table 4:** Contrast statements to compare herbicide programs for density and aboveground biomass of glyphosate-resistant giant ragweed and crop yield in glyphosate plus glufosinate-resistant maize in a field experiment conducted in 2013 and 2014 in Nebraska, United States.^a^

Treatment	Giant ragweed^b^		Maize yield^b^	
	Density (# m^-1^)	Biomass (g m^-1^)	2013 (kg ha^-1^)	2014 (kg ha^-1^)
2,4-D vs. dicamba	8 vs. 2^∗^	63 vs. 89^∗∗^	3,619 vs. 8,068^∗∗^	8,243 vs. 11,286^∗∗^
Glufosinate + 2,4-D vs. 2,4-D alone	3 vs. 8^∗^	80 vs. 63^∗∗^	5,591 vs. 3,619 NS	10,457 vs. 8,243^∗∗^
Glufosinate + 2,4-D vs. glufosinate alone	3 vs. 4 NS	80 vs. 78 NS	5,591 vs. 6,344 NS	10,457 vs. 8,695^∗^
Glufosinate + dicamba vs. dicamba alone	2 vs. 2 NS	92 vs. 95 NS	9,749 vs. 8,068 NS	10,844 vs. 11,286 NS
Glufosinate + dicamba vs. glufosinate alone	2 vs. 4 NS	92 vs. 78^∗^	9,749 vs. 6,344^∗^	10,844 vs. 8,695^∗∗^
Glufosinate + 2,4-D + dicamba vs. glufosinate + 2,4-D	3 vs. 3 NS	84 vs. 80 NS	7,163 vs. 5,591 NS	10,143 vs. 10,457 NS
Glufosinate + 2,4-D + dicamba vs. glufosinate + dicamba	3 vs. 2 NS	84 vs. 92 NS	7,163 vs. 9,749 NS	10,143 vs. 10,844 NS

*^a^Preplanned single degree of freedom contrast statements were used to compare treatments with herbicides used alone (2,4-D, dicamba or glufosinate) versus treatment combinations of 2,4-D and/or dicamba plus glufosinate, and three-way versus two-way combinations of 2,4-D, dicamba and glufosinate. ^b∗^Significant with *P* ≤ 0.05; ^∗∗^significant with *P* ≤ 0.01, NS, non-significant or *P* ≥ 0.05.*

### Maize Injury and Yield

Herbicide treatments including 2,4-D and dicamba alone at the higher rate (560 g ae ha^-1^) or tank-mixed with glufosinate resulted in 2 to 12% maize injury at 14 DAT (data not shown); however, the injuries were transient and did not result in yield reduction. Maize yields were lower under rainfed conditions in 2013 compared to irrigated conditions in 2014; therefore, yield data were presented separately for both years. Among herbicide treatments, 2,4-D at 280 g ae ha^-1^ resulted in the lowest maize yield in both years (**Table 3**). Glufosinate (450 g ai ha^-1^) plus dicamba (560 g ae ha^-1^) resulted in the highest maize yield (10,783 and 12,416 kg ha^-1^ in 2013 and 2014), respectively; however, the yield was comparable among most of herbicide treatments with the exception of glufosinate at 450 g ai ha^-1^ plus 2,4-D at 280 g ae ha^-1^ (5,035 kg ha^-1^) in 2013 and glufosinate at 450 g ai ha^-1^ (7,246 kg ha^-1^) in 2014 (**Table 3**). Contrast analysis of yield in 2013 suggested that average maize yield with glufosinate plus dicamba was 9,749 kg ha^-1^ compared to the average yield (6,344 kg ha^-1^) with glufosinate applied alone. Similarly, contrasts between 2,4-D vs. dicamba were also significant (*P* ≤ 0.01), while all other contrast statements were non-significant (*P* > 0.05) (**Table 4**). In 2014, however, the contrast statements, including 2,4-D vs. dicamba, glufosinate plus 2,4-D vs. 2,4-D or glufosinate, and glufosinate plus dicamba vs. glufosinate, were significant (*P* ≤ 0.05) (**Table 4**).

## Discussion

Giant ragweed control with 2,4-D or dicamba was in consensus with previous researches. For example, Barnett and Steckel (2013) reported 47 and 64% control of GR giant ragweed at 10 DAT with 2,4-D applied at 560 and 1,120 g ae ha^-1^, respectively. In contrast, Kaur et al. (2014) reported 98% control of GR giant ragweed with 2,4-D at 560 g ae ha^-1^ applied 21 days before planting soybean when giant ragweed was ≤6 cm tall, compared with this study, where giant ragweed was 8 to 12 cm tall at the time of POST herbicide application. Similarly, Barnett and Steckel (2013) reported 62 to 67% control of GR giant ragweed at 10 DAT with dicamba applied at 280 or 560 g ae ha^-1^. Irrespective of the application rate, dicamba provided a better giant ragweed control compared to 2,4-D. This might be because giant ragweed is more sensitive to dicamba compared to 2,4-D, though this might not be the case for other weed species. For example, Meyer et al. (2015) reported no differences between 2,4-D- and dicamba-based programs for the control of Palmer amaranth and common waterhemp. Glufosinate applied alone at 590 g ai ha^-1^ or tank-mixed with 2,4-D and/or dicamba provided ≥80% giant ragweed control. Similarly, Norsworthy et al. (2010) reported ≥90% control of GR or GS giant ragweed with glufosinate at 590 g ai ha^-1^ irrespective of growth stage at the time of application. Likewise, Craigmyle et al. (2013b) reported that tank-mixing 2,4-D amine at 560, 840, or 1,120 g ae ha^-1^ with glufosinate improved common waterhemp control to ≥95% compared with 75 to 92% or 78 to 98% control following 2,4-D or glufosinate, respectively.

Increasing 2,4-D rate from 280 to 560 g ae ha^-1^ improved giant ragweed control from ≤30% to 56 to 73%. Previous research also reported that increasing 2,4-D rate improved broadleaf weed control (Everitt and Keeling, 2007; Sarabi et al., 2011). For instance, 2,4-D provided less than 80% control of redroot pigweed (*Amaranthus retroflexus* L.) when applied at 400 g ae ha^-1^ compared to 100% control at 600 to 1,000 g ae ha^-1^ (Sarabi et al., 2011). However, dicamba or glufosinate resulted in comparable giant ragweed control throughout the season regardless of the rate of application. Soltani et al. (2011) reported 90% giant ragweed control at 56 DAT with dicamba at 600 g ae ha^-1^. Nevertheless, Craigmyle et al. (2013b) reported that control of 20 to 25 cm tall common waterhemp improved from 84 to 90% with increasing glufosinate application rate from 450 to 730 g ai ha^-1^. The results of this study indicated an additive interaction between glufosinate plus 2,4-D and/or dicamba. However, Joseph (2014) reported synergistic interaction between glufosinate plus dicamba for control of sicklepod [*Senna obtusifolia* (L.) Irwin and Barneby]. Steckel et al. (2006) reported at least 90% horseweed [*Conyza canadensis* (L.) Cronq.] control with application of glufosinate (470 g ai ha^-1^) plus 2,4-D (530 g ae ha^-1^) or dicamba (280 g ae ha^-1^) at 14 and 56 DAT.

As with the results of giant ragweed control, the results of giant ragweed density and biomass reduction were in agreement with previous studies. Barnett and Steckel (2013) reported 5.8 and 7.3 giant ragweed plants m^-2^ with glufosinate and 2,4-D (560 g ai ha^-1^). Chahal and Johnson (2012) reported comparable biomass reduction in horseweed and common lambsquarters (*Chenopodium album* L.) with glufosinate or glufosinate plus 2,4-D or dicamba. However, Barnett and Steckel (2013) reported a biomass of 19 and 23 g m^-2^ with glufosinate (590 g ai ha^-1^) and 2,4-D (560 g ae ha^-1^) compared to ≤12.5 g m^-2^ with 2,4-D applied at 1,120 g ae ha^-1^, dicamba (280 or 560 g ae ha^-1^), and glufosinate plus 2,4-D or dicamba irrespective of the application rate.

The results of this study revealed that 2,4-D (280 or 560 g ae ha^-1^) resulted in ≤73% giant ragweed control throughout the season. Dicamba (280 or 560 g ae ha^-1^) initially provided ≤83% control, but the control improved to ≥95% by 60 DAT or at harvest. The improvement in the efficacy of dicamba occurred most likely due to its systemic nature. In contrast, glufosinate initially resulted in 85 to 92% giant ragweed control, but control declined to 70 to 79% and 83 to 87% with 450 and 590 g ae ha^-1^ of glufosinate, respectively, at 60 DAT or at harvest. Similarly, Jhala et al. (2013) reported reduced control of Brazil pusley (*Richardia brasiliensis* Moq.), puncture vine (*Tribulus terrestris* L.), and eclipta (*Eclipta prostrata* L.) with glufosinate at 30 DAT compared to 15 DAT. Reduction in control of giant ragweed following glufosinate at 60 DAT compared to 14 or 28 DAT may be attributed to its contact nature and lack of residual activity (Anonymous, 2016). Glufosinate plus 2,4-D or dicamba provided 91 to 97% giant ragweed control at 14 and 28 DAT; nevertheless, control at 60 DAT and at harvest ranged from 80 to 92% with glufosinate plus 2,4-D in contrast to 89 to 98% control with glufosinate plus dicamba. Likewise, glufosinate plus dicamba plus 2,4-D provided more than 90% giant ragweed control throughout the season (**Table 1**). The herbicide mixtures showed an additive interaction at the rates used in this study, suggesting that mixtures including glufosinate plus 2,4-D or dicamba resulted in greater or mostly comparable giant ragweed control and reduction in density or aboveground biomass compared to when applied alone (**Table 1**). Similarly, Barnett and Steckel (2013) and Craigmyle et al. (2013a,b) reported an improved efficacy of glufosinate for control of giant ragweed and common waterhemp, respectively, when tank-mixed with synthetic auxins (2,4-D or dicamba) compared to glufosinate applied alone. Studies have also reported that glufosinate plus dicamba applied as PRE, early-post (EPOST), or mid-post (MPOST) improved control (79 to 100%) of Palmer amaranth compared to glufosinate alone (72 to 90%) (Cahoon et al., 2015). However, the interactions between the herbicides in a mixture may vary with the active ingredient, the weed species, and even the rate of the respective herbicides in a mixture. For example, synergistic interactions have been reported with 2,4-D plus halosulfuron for common lambsquarters control (Isaacs et al., 2006), and mesotrione plus glufosinate for common ragweed (*Ambrosia artemisiifolia* L.) and giant foxtail (*Setaria faberi* Herrm.) control (Armel et al., 2008). Conversely, Burke et al. (2005) reported that glufosinate at 290 or 410 g ai ha^-1^ antagonized clethodim, resulting in a reduction of goosegrass [*Eleusine indica* (L) Gaertn] control from ≥90% to ≤40%. Similarly, Koger et al. (2007) reported antagonistic effects of monosodium methanearsonate (MSMA) on glufosinate efficacy in browntop millet, hemp sesbania, ivyleaf morningglory [*Ipomoea hederacea* (L.) Jacq.], johnsongrass [*Sorghum halepense* (L.) Pers.], Palmer amaranth, pitted morningglory (*Ipomoea lacunosa* L.), prickly sida (*Sida spinosa* L.), redroot pigweed, and velvetleaf (*Abutilon theophrasti* Medik.). Therefore, though additive interactions between glufosinate and 2,4-D or dicamba were observed in GR giant ragweed, those interactions may vary with other weed species or tank-mix partners, including differing rates of 2,4-D or dicamba with glufosinate not tested in this study.

## Conclusion

Results of this study indicated that tank-mixing glufosinate with 2,4-D or dicamba showed an additive interaction and provided an effective POST option for the control of GR giant ragweed in maize and secured optimum yield. Although results of this study reported excellent control of giant ragweed with 2,4-D/dicamba tank-mixed with glufosinate, a diverse weed management program should be adopted by growers, because relying on these herbicides, particularly applied alone, may result in the evolution of resistant weeds. For example, 2,4-D-resistant common waterhemp in eastern Nebraska (Bernards et al., 2012) and dicamba-resistant kochia in western Nebraska (Crespo et al., 2014) have been confirmed. Similarly, glufosinate resistance has been reported in few weed species including goosegrass, Italian ryegrass [*Lolium perenne* L. ssp. *multiflorum* (Lam.) Husnot], and perennial ryegrass (*Lolium perenne* L.) (Jalaludin et al., 2017). Thus, over-reliance on 2,4-D, glufosinate, or dicamba should be avoided and a diversity of herbicide chemistries must be maintained by using herbicide tank-mixtures with multiple effective modes of action, along with non-chemical weed control methods including crop rotation, tillage, competitive cultivars, weed seed destruction, and cover crops, among others (Norsworthy et al., 2012).

The rapid evolution of GR weeds has emphasized the importance of diverse weed management approaches, including PRE followed by POST herbicide programs along with non-chemical methods (Norsworthy et al., 2012; Riley and Bradley, 2014). Therefore, to avoid overdependence on these herbicide mixtures and ensure an effective use of multiple-resistant crop technology without enhancing the evolution of multiple herbicide-resistant weeds, an integrated weed management approach for GR giant ragweed or other weed species should be implemented. Recently, integrated weed management approaches involving preplant tillage followed by PRE and/or POST herbicides with multiple modes of action have been developed for the effective management of GR giant ragweed in maize and soybean (Ganie et al., 2016, 2017). Future studies should consider the evaluation of these herbicide mixtures for the control of other prominent GR weed species including common ragweed, common waterhemp, horseweed, kochia (*Kochia scoparia* L.), and Palmer amaranth in Nebraska.

## Author Contributions

ZG conducted the experiments, analyzed the data and wrote the manuscript and AJ conceptualized and designed the research and edited manuscript.

## Conflict of Interest Statement

The authors declare that the research was conducted in the absence of any commercial or financial relationships that could be construed as a potential conflict of interest.

## Footnotes

1 http://www.R-project.org
